# Navigate Towards the Immunotherapy Era: Value of Immune Checkpoint Inhibitors in Non-Small Cell Lung Cancer Patients With Brain Metastases

**DOI:** 10.3389/fimmu.2022.852811

**Published:** 2022-03-29

**Authors:** Guanqun Yang, Ligang Xing, Xiaorong Sun

**Affiliations:** ^1^ Cheeloo College of Medicine, Shandong University, Jinan, China; ^2^ Department of Radiation Oncology, Shandong Cancer Hospital and Institute, Shandong First Medical University and Shandong Academy of Medical Sciences, Jinan, China; ^3^ Department of Nuclear Medicine, Shandong Cancer Hospital and Institute, Shandong First Medical University and Shandong Academy of Medical Sciences, Jinan, China

**Keywords:** brain metastases (BMs), non-small cell lung cancer (NSCLC), immune checkpoint inhibitors (ICI), combination strategies, patient selection, response assessment

## Abstract

Brain metastases (BMs) in non-small-cell lung cancer (NSCLC) patients are associated with significant morbidity and poor prognosis. Immune checkpoint inhibitors (ICIs) have resulted in a paradigm shift in the management of advanced NSCLC. However, the value of ICIs in NSCLC patients with BMs remains unclear because patients with BMs are routinely excluded in numerous prospective trials on ICIs. Here, starting from the mechanisms of ICIs for BMs, we will reveal the value of ICIs by reviewing the efficacy and adverse effects of ICIs monotherapy as well as promising combination strategies, such as combinations with chemotherapy, radiotherapy, and anti-angiogenic drugs, etc. In addition, the methods of patient selection and response assessment will be summarized to assist clinical practice and further studies.

## Introduction

Brain metastases (BMs) are frequent complications in patients with non-small cell lung cancer (NSCLC), present in 10% ~ 20% of patients at diagnosis, and approximately 20% ~ 40% eventually (1, 2). The incidence of NSCLC BMs is increasing, partly due to the improvements in testing techniques and the popularity of screening, as well as the improvements in therapies that extend patient survival (3).

NSCLC-BMs are associated with poor prognosis (4). In patients with driver-gene positive NSCLC-BMs, such as those harboring epidermal growth factor receptor (*EGFR*) mutation or anaplastic lymphoma kinase (*ALK*) rearrangement, new-generation targeting reagents have a favorable intracranial response rate (66%–78%) (5, 6). However, treatments for those driver-gene negative patients are extremely limited. Radiotherapy is the mainstream treatment for symptomatic BMs, such as whole brain radiotherapy (WBRT) and stereotactic radiation therapy (SRT). Surgical resection is only appropriate for a limited number of carefully selected patients. Management of multiple asymptomatic BMs often involves systemic therapy only. However, conventional systemic therapies could not achieve desired intracranial efficacy and survival improvements (7). Overall, platinum-based chemotherapy has obtained 30%~40% intracranial response, while the benefit is short-lasting and the toxicity is enormous (8). Hence, optimization of the treatment of NSCLC-BMs is urgently needed.

Blocking the programmed death protein-1 (PD-1)/its ligand (PD-L1) axis with immune checkpoint inhibitors (ICIs) has revolutionized the treatment landscape for advanced NSCLC, covering from first-line treatment to post-line treatment (9). In addition, ICIs in combination with chemotherapy or radiotherapy were approved for metastatic NSCLC. Cytotoxic T lymphocyte-associated antigen-4 (CTLA-4) inhibitors, such as ipilimumab, have a good performance in combination treatment, despite poor performance as monotherapy for NSCLC. Unfortunately, the value of ICIs for NSCLC-BMs is indeterminate because those patients have generally been excluded or underrepresented in over half of the clinical trials (10). There are several reasons for this issue: (1) The poor survival of patients with BMs may increase the probability of trial failure (11); (2) Drugs are difficult to penetrate through the blood-brain barrier (BBB) to intracranial lesions (12); (3) The tumor microenvironment of BMs is immunologically “cold” (13); (4) Patients with symptomatic BMs often need steroids, which may conflict with immunotherapy (14). Although some clinical trials enrolled asymptomatic BMs, the outcomes of the BMs subgroup were rarely reported (10). Until recently, several clinical trials of ICI treatment for advanced NSCLC, especially combination therapy, have published the results containing BMs subgroup analysis. Although the evidence is still limited, NSCLC-BMs are navigating towards the era of immunotherapy.

The main aim of this review is to reveal the value of ICIs for NSCLC-BMs. Based on the possible mechanisms through which BMs can benefit from ICIs, we summarize clinical evidence, including pivotal prospective trials and representative studies. Future challenges and perspectives will also be sketched out in order to better understand and optimize ICI-containing treatments in patients with BMs.

## Mechanisms

Historically, the physiological brain was regarded as an immune-privileged organ, mainly due to the BBB and genetically special immune spectrum (15). However, the mechanism of ICIs, unlike targeted-tumor cell drugs, theoretically relates to modified immune cell activity rather than a direct action of tumor cells in the brain. Moreover, changes in the neuroimmunology background have rekindled interests in immunotherapy for BMs.

It was generally believed that molecules with large molecular weight and low liposolubility, as well as peripheral immune cells, cannot penetrate the BBB (16). Actually, the existence of BMs and the management of anti-tumor treatments can lead BBB to become “not too dense” (17, 18). The BBB is induced to structural changes and dysfunction by BMs, and possible mechanisms include the increase in vascular endothelial growth factor (VEGF)-mediated angiogenesis and multiple adhesion molecules (such as VCAM-1 and ICAM-1) and chemokines (such as CXCL12-CSCR4 axis)-mediated trans-endothelial migration (18). Indeed recently, nivolumab has been measured in cerebrospinal fluid (CSF) of five patients with suspected leptomeningeal metastases, with CSF/plasma ratios ranging from 1/52 to 1/299 (19). Furthermore, radiotherapy can loosen the BBB. After brain radiotherapy, the CSF/plasma ratio of trastuzumab could increase by six times (20). Unfortunately, pharmacokinetic studies of ICIs after radiotherapy are absent. Collectively, during the development and treatment of BMs, the tight fences of BBB may be opened.

A deeper understanding of the tumor microenvironment (TME) is necessary to develop immunotherapy (21). Tumor infiltrating lymphocytes (TILs) are essential for the efficacy of immunotherapy. Other immune cells in the brain TME, such as tumor-associated macrophages (TAMs), microglia, and astrocytes that surround brain tumors are involved in tumor progression and immune evasion (22). Whether lymphocytes can cross the BBB had remained controversial for decades. Recent studies have refuted the notion of immune isolation in the brain. In the 1980s, an antigen exit route from the brain to the deep cervical lymph nodes was discovered (23). In 2015, functional lymph-vessel found in the meninges provided a direct drainage route for immune cells from the brain to cervical lymph nodes (24). Despite the discovery of T cell infiltration in primary brain tumors (25), outcomes from PD-1/PD-L1 ICB trials in gliomas are disappointing to date (26). It is partially attributed to insufficiency of TILs infiltration, low expression of PD-1/PD-L1, and low tumor mutation burden (TMB) in gliomas (26), which are not conducive to the ICIs to revive the anti-tumor immune response. Compared with gliomas, however, BMs are more abundant and diverse with TILs and neutrophils (27). Relatively high infiltration of TILs has been found in BMs from melanoma, renal cell carcinoma, and NSCLC (28). Comparing PD-L1 expression and TILs densities between primary tumor and matched BMs revealed a lower burden of TILs but a higher PD-L1 expression in NSCLC BMs (29–31). Given the discordance between BMs and primary tumors, these differences may contribute to the differential activity of immunotherapy for NSCLC-BMs. The intracranial efficacy of pembrolizumab was confirmed despite the relative scarcity of intracranial TILs (32). Hence, it is necessary to determine whether the intracranial efficacy of ICIs depends on activation of intracranial immune cells in situ, or migrating from the peripheral environment, or both. Using a high-dimensional single-cell approach, Friebel et al. revealed that BMs were characterized by high infiltration of peripherally-derived leukocytes, especially CD8+ T cells (33). Moreover, CD8+ T cells in BMs appear to be more exhausted than those in peripheral and normal intracranial environments, partially because immune suppressive signals, such as PD-1 and CTLA-4, are upregulated, a mechanism that ICIs can potentially improve. Studies have shown that blocking PD-1 could induce the migration of immune cells to the brain, in which IFN-γ played a key role (34). Up-regulation of IFN-γ can modulate multiple adhesion molecules (such as VCAM-1 and ICAM-1) and chemokines (such as CXCL10)-mediated T cell migration and can also induce the turning on of BBB (35, 36). Notably, activated CD4+ T cells in the brain can loosen the BBB through local IFN-γ production (37), which may produce positive feedback.

**Figure 1:**
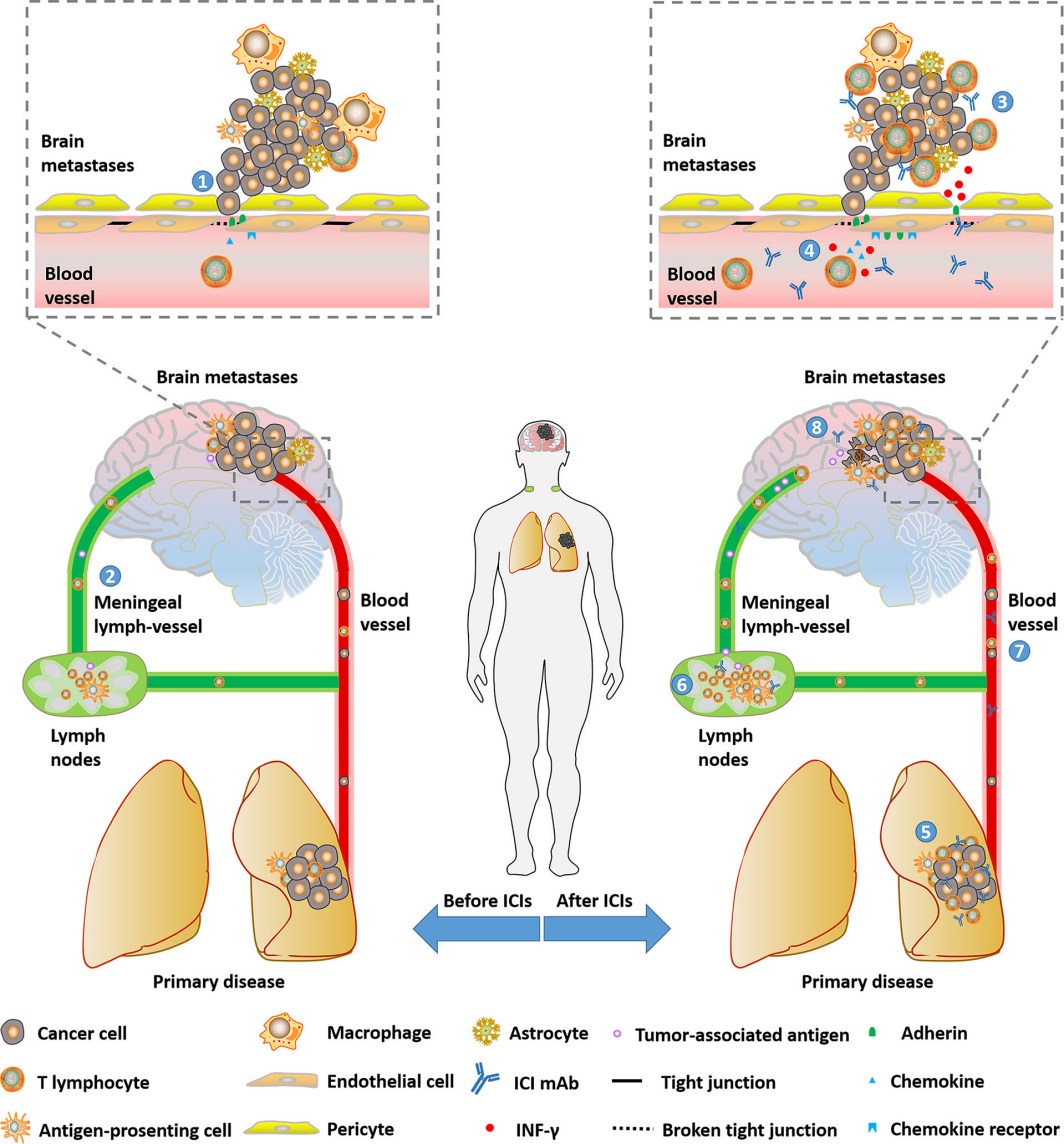
Potential mechanisms that NSCLC patients with brain metastases could benefit from ICIs. (1) BBB will be loosened with the progression of BMs. (2) Immune cells and tumor-associated antigen could be transported between intracranial and peripheral environments by meningeal lymph-vessels. (3) Part of ICI mAbs can enter the intracranial environment and be detected. (4) BBB can get loose due to broken tight conjunction and enhanced trans-endothelial migration through the up-regulation of adhesins and chemokines mediated by INF-γ inducing T-cell- secreted INF-γ. (6)(7)(8) ICIs revive anti-tumor immune in lymph nodes, primary disease and peripheral circulation. ICI, immune checkpoint inhibitor; RT, radiotherapy; CT, chemotherapy; IFN, interferon; BBB, blood-brain barrier; mAb, monoclonal antibody; IFN, interferon.

In conclusion, these findings indicated that NSCLC patients with BMs might benefit from ICIs treatment by activated intracranial and extracranial immune (**Figure 1**).

## ICI Monotherapy for NSCLC-BMs

### Response

For a long time, it has been controversial whether ICI monotherapy induces an intracranial response in NSCLC-BMs. Intracranial examinations were routinely not been performed in the follow-up when observing the response of ICIs for advanced NSCLC. Several large randomized trials on ICIs treatment for gliomas (Checkmate-143, Checkmate-498, Checkmate-548) failed to prolong PFS and OS (26), casting a shadow over research on metastatic brain tumors. This nebulous status continued until Goldberg and his colleagues firstly reported the outcome of pembrolizumab for patients with untreated BMs (32). In their phase 2 trial, patients with at least one 5~20mm untreated asymptomatic BMs were divided into two cohorts. Cohort 1 was patients with PD-L1 positive (PD-L1≥1%) and cohort 2 PD-L1 negative (PD-L1<1%) or unknown. The results showed that 11 of 37 patients (29.7%) in cohort 1 had an intracranial response. However, there was no response in cohort 2 (5 patients). Furthermore, 29.7% of patients in cohort 1 had a systemic response, and 7 of the intracranial responders had a systemic response simultaneously. Several retrospective studies also provided valuable evaluations of intracranial response (38–43). In short, intracranial objective response rate (ORR) ranges from 16.4% to 36.6% regardless of PD-L1 expression. For patients with PD-L1≥50%, intracranial ORR may exceed 50%.

Compared with chemotherapy, a higher systemic response has been observed in ICI monotherapy for patients with BMs. Recently, a pooled analysis based on KEYNOTE-001, 010, 024, and 042 (44) showed that systemic ORR with pembrolizumab was superior to chemotherapy in PD-L1≥1% NSCLC patients with BMs (26.1% vs. 18.1%). The single-arm FIR trial (45) enrolled 13 advanced NSCLC patients treated with atezolizumab into its cohort 3 (second-line with treated BMs). All eligible patients were PD-L1 positive. Investigator-assessed systemic ORR was 23%. Of particular interest, the outcome of FIR trial as well as other retrospective data suggested that there was no significant difference between patients with asymptomatic BMs and patients without BMs (45) (**Table 1**).

**Table 1:** Clinical investigations of ICI monotherapy in the treatment of NSCLC patients with BMs.

Data source	Arm (patients with BMs/all patients)		BMs Condition	PD-L1	Response (ORR)		Survival		Safety (≥3 Grade AEs)	
	Experimental	Control			CNS	Systemic	PFS	OS	CNS	Systemic
Prospective (32)	Pembro (42).	N/A	Asymptomatic; Untreated; 4~20 mm	≥1%	29.7%	29.7%	1.9m	9.9m	0%	14%
				<1%	0.0%	NR	NR	NR		
Prospective (44)	Pembro. (199/1753)	CT (94/1217)	Asymptomatic	≥50%	NR	33.9% vs. 4.6%	4.1m vs. 4.6m	19.7m vs. 9.7m	9.7% vs. 26.7%;	14.8% vs. 45.6%
				≥1%	NR	26.1% vs. 18.1%	2.3m vs. 5.2	13.4m vs. 10.3m		
Retrospective (38)	Pembro. in BMs (126/547)	Pembro. in non-BMs (444/570)	NR	NR	36.4%	27.8% vs. 29.7%	9.2m vs. 7.7m	18.0m vs. 18.7m	NR	NR
Retrospective (40)	Pembro. in BMs (23/87)	Pembro. in non-BMs (64/87)	NR	≥50%	70%	NR	6.5m vs. 7.0m	21.6m vs. 24.6m	NR	23% vs. 30%
Prospective (46)	Nivo. (45/427)	CT (42/427)	Asymptomatic; Treated	NR	NR	NR	NR	7.6m vs. 6.2m	NR	NR
RWS (47)	Nivo. in BMs (1800/10452)	Nivo. in non-BMs (8652/10452)	NR	NR	NR	NR	NR	9.9m vs. 12.1m	NR	NR
RWS (48)	Nivo. (477/2585)	N/A	Treated	NR	NR	NR	NR	9.7m	0%	NR
RWS (49–51)	Nivo. (446/1959)	N/A	Asymptomatic; Nonsquamous	NR	NR	17%	3.0m	8.6m	NR	7%
			Asymptomatic; Squamous;	NR	NR	19%	4.9m	5.8m	NR	8%
Retrospective (41)	Nivo. in BMs (32/73)	Nivo. in non-BMs (41/73)	NR	NR	28.1%	25.0% vs. 19.5%	2.8 m vs. 4.9m	14.8m vs. 20.29m	NR	NR
Prospective (45)	Atezo. (13/137)	N/A	Asymptomatic; Treated	All	NR	23%	2.5m	6.8m	NR	15%
			≥50%	NR	25%	2.3 m	7.0m	NR	NR
Prospective (52)	Atezo. (61/425)	CT (62/425)	Asymptomatic; Treated	NR	NR	NR	NR	16.0m vs. 11.9m, HR=0.74	5.0% vs. 1.8%	23.3% vs. 50.9%
Prospective (53)	Cemip. (34/283)	CT (34/280)	Asymptomatic; Treated	≥50%	NR	NR	HR=0.45	HR=0.17	NR	NR
Retrospective (54)	ICI (840/1680)	Non-ICI (840/1680)	NR	NR	NR	NR	NR	12.8m vs. 10.1m, HR=0.80	NR	NR
RWS (43)	ICI (41)	N/A	NR	NR	36.6%	24.4%	6.2m	13.7m	NR	NR

NSCLC, Non-Small Cell Lung Cancer; BMs, Brain Metastases; AEs, Adverse Events; N/A, Not Applicable; NR, Not Reported; nr, Not Reach; HR, Hazard Ratio; RWS, Real-word Study; ICI, immune checkpoint inhibitor; CT, chemotherapy; Pembro., Pembrolizumab; Nivo., Nivolumab; Atezo., Atezolizumab; Cemip., Cemiplimab; OS, Overall Survival; PFS, Progression-free Survival; ORR, Objective Response Rate; CNS, central nervous system.

### Survival

Benefits in survival are relatively clear-cut for NSCLC patients with BMs. A pooled analysis of KEYNOTE-001, 010, 024, and 042 (44) showed that pembrolizumab improved overall survival (OS) versus chemotherapy (13.4 months vs. 10.3 months, HR=0.83). In the subgroup of NSCLC patients with high PD-L1 expression (PD-L1≥50%), the magnitude of benefit with pembrolizumab compared with chemotherapy increased to 19.7 months vs. 9.7 months (HR=0.67). Importantly, both the magnitude of benefit and the toxicity profile with pembrolizumab were similar to those in patients without BMs (38, 40). A pooled analysis of CheckMate-017 and 057 (46) compared the long-term outcomes of nivolumab with docetaxel in the BMs subgroup. Nivolumab improved OS (7.6 months vs. 6.2 months, HR=0.81) and 5-year OS rate (8% vs. 0%) versus docetaxel. Clinical evidence that NSCLC patients with BMs can benefit from nivolumab mainly came from several real-world studies in European regions (41, 47–51). Several retrospective studies that did not distinguish the types of anti-PD-1/PD-L1 monotherapy were also shown in **Table 1**. Cemiplimab is a newly approved anti-PD-1 ICI for advanced NSCLC with high PD-L1 expression(≥50%) based on a phase 3 EMPOWER-Lung 1 study (53). Results have demonstrated that cemiplimab monotherapy present significantly superior progression-free survival (PFS) (HR=0.45) and OS (HR=0.17) compared with platinum-double chemotherapy in the BMs subgroup. A similar benefit could be observed in OAK trials comparing atezolizumab with docetaxel (52). Notably, the development of new BMs was delayed by atezolizumab. In patients with baseline BMs, the median time to the development of new BMs was not reached in the atezolizumab arm, and was 9.3 months in the docetaxel arm (HR=0.38). Interestingly, in PACIFIC study for inoperable stage 3 NSCLC patients (55), the maintenance treatment of durvalumab after chemoradiotherapy was associated with a halved incidence of developing new BMs. These findings indicated PD-L1 inhibitors might prevent or at least delay the occurrence of BMs.

### Safety

According to the published data, it was generally accepted that ICI monotherapy had a better tolerance than standard chemotherapy. The prospective trial focusing on BMs from Goldberg et al. (32) reported that no intracranial Grade≥3 adverse events (AEs) occurred, and the incidence of systemic Grade≥3 AEs was 14%. In the pooled analysis of KEYNOTE-001, 010, 024, and 042 (44), comparing pembrolizumab and chemotherapy, the incidence of intracranial Grade≥3 AEs was 9.7% vs. 26.7%, while the incidence of systemic Grade≥3 AEs was 14.8% vs. 45.6%. The Expanded Access Program from Italy included 466 NSCLC patients with BMs who were asymptomatic after radiotherapy. 7% nonsquamous NSCLC patients and 8% squamous NSCLC patients suffered Grade≥3 systemic AEs (50, 51). Another real-world study reported similar incidence, regardless of neither histological type nor condition of BMs (56). The BMs subgroup analysis from OAK trial (52) indicated the better systemic safety of atezolizumab compared with docetaxel. Notably, Grade≥3 neurologic treatment-related AEs (trAEs) were higher in the atezolizumab arm, although the rate of neurocognitive AEs was quite low in both arms (5.0% vs. 1.8%), and no Grade 4~5 neurologic trAEs occurred. Collectively, the incidence of Grade≥3 trAEs ranges from 14%~25%, as well as a low incidence of intracranial AEs.

To sum up, anti-PD-1/PD-L1 ICI monotherapy is beneficial for NSCLC patients with BMs, which can diminish intracranial disease, reduce adverse events and improve survival, particularly for those with high PD-L1 expression (**Table 1**). However, we still need more large-scale clinical data to support this view and precisely pre-stratify patients who may benefit.

## Combination Strategy for NSCLC-BMs

**Figure 2:**
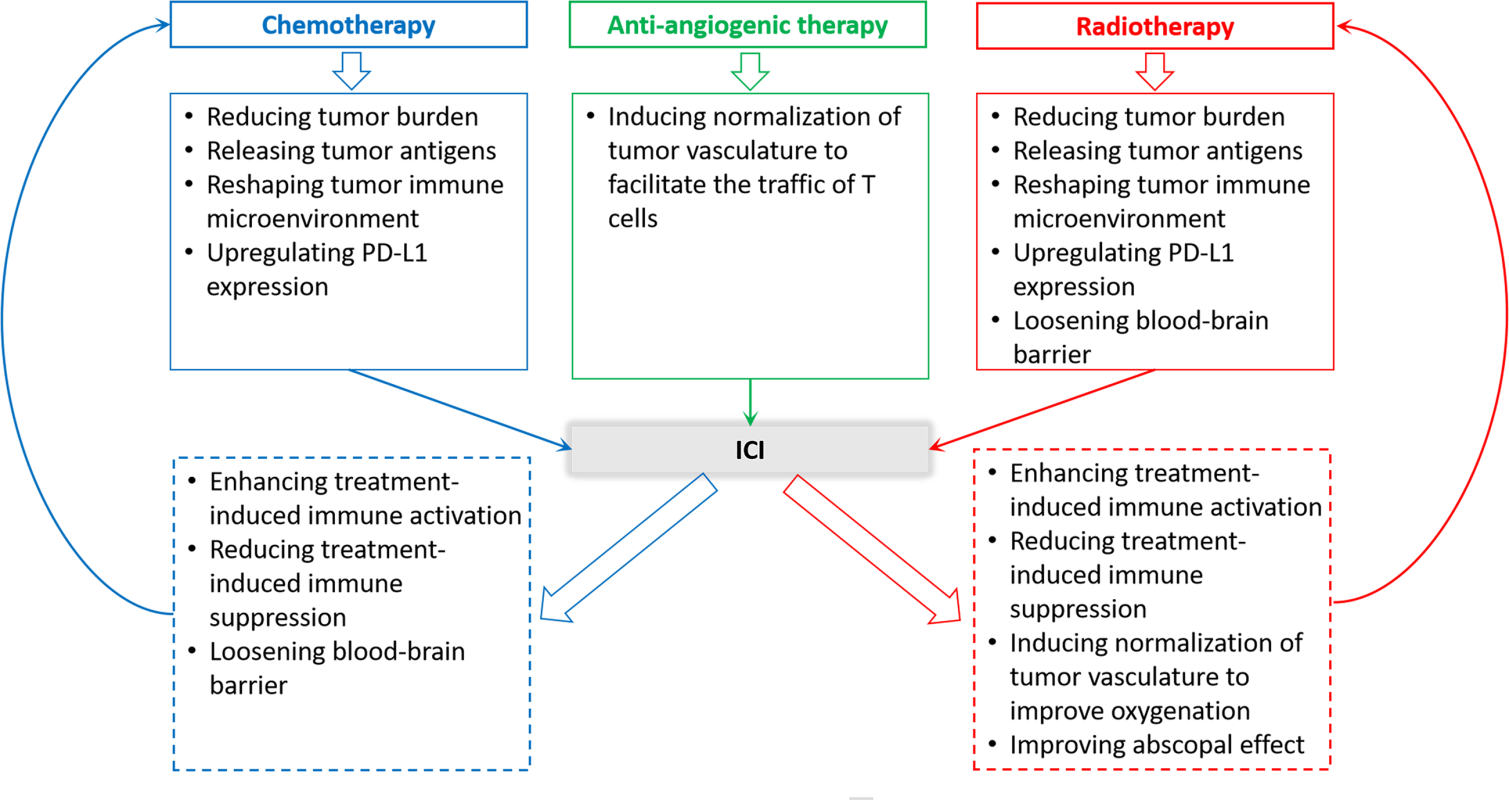
Rationality of ICI therapy and other therapies combination in the treatment of NSCLC patients with BMs.

Although ICI monotherapy could yield benefits to NSCLC patients with BMs, the response of ICI monotherapy is generally less than 30%. Moreover, due to the slow onset of the anti-tumor immune response, there is often a cross point on the Kaplan-Meier survival curves between ICI monotherapy group and the control group when curves are priming, suggesting that the early efficacy of ICI treatment was inferior to chemotherapy or radiotherapy for some patients. The outcomes of the Keynote-001 study showed that about 75% of patients who produced early response on immunotherapy achieved long-term survival (57). Hence, improving ICI efficacy and expanding the pool of beneficiaries are urgently needed. Actually, immunotherapy can be reciprocally beneficial to other treatments (**Figure 2**), such as radiotherapy and chemotherapy.

### ICI Combined With Radiotherapy

Given almost all patients with metastatic NSCLC require radiotherapy, especially those with BMs, radiotherapy and immunotherapy possibly form the best alliance in clinical practice. On the one hand, a growing body of evidence supports synergistic mechanisms between radiotherapy and immunotherapy (58–60), which are present in **Figure 2**. On the other hand, from a clinical point of view, the response of immunotherapy is slow but persistent, whereas radiotherapy can quickly relieve nervous symptoms in BMs patients, although the response is not long-lasting (60). Therefore, combination treatment can bring effective and long-term benefits to BMs patients from NSCLC.

Some clinical studies have provided support for the value of this alliance. ICIs combined with radiotherapy can convey benefits to NSCLC patients with BMs by elevation in intracranial disease control, prolongation survival as well as improvement in neurocognitive function. A multicenter phase II trial (61) examined the effect of combining nivolumab with SRT. It included 26 patients (22 NSCLC), having ≤10 cc of BMs with no prior irradiation or immunotherapy. Of the 21 NSCLC patients with assessable PD-L1 status, 12 patients had PD-L1≥50%. High intracranial control was present: median intracranial PFS was 5.0 months, the 1-year cumulative incidence of intracranial relapse was 20% accounting for death as a competing risk. Median OS was 14 months. In the sixth month, neurocognitive function showed potential improvements. Compared with radiotherapy alone, radiotherapy plus ICI significantly improved local disease control and survival. A representative example is an investigation with a large sample size from National Cancer Databank (NCDB), showing that the median OS of NSCLC-BMs patients treated with ICI+RT increased 1.5 times (13.1 months vs. 9.7 months) than those treated with RT alone (62). Moreover, combination therapy can decrease neurological death (63). More data are listed in **Table 2**.

**Table 2:** Clinical investigations of ICI and radiotherapy combination in the treatment of NSCLC patients with BMs.

Data source	Arm (patients with BMs/all patients)			BMs Condition	Response (LNR)		Survival	Safety (≥ 3 Grade AEs)	
	Experimental		Control		CNS	Systematic		CNS	Systematic
Prospective (61)	Concurrent (within 2 weeks) SRT after the first dose of Nivo (26).		N/A	Untreated; ≤ 10 cc;	80%	51.7%	iPFS: 5.0m; OS: 14m	NR	NR
Retrospective (62)	RT before or after ICI (545/14090)		RT alone (13545/14090)	NR	NR	NR	OS: 13.1m vs. 9.7m	NR	NR
Retrospective (63)	SRT before or after ICI (within 3 months) (33/77)		SRT alone (44/77)	NR	97% vs. 86%	NR	OS: HR = 0.46	NR	NR
Retrospective (64)	Concurrent (within 4 weeks) ICI after SRT (100/150)		SRT alone (50/150)	≤4 cm;	NR	NR	iPFS: HR = 0.32	NR	NR
Retrospective (65)	SRT before or after ICI (within 3 months) (17/51)		SRT alone (34/51)	NR	84.9% vs. 76.3%	NR	NR	5.9% vs. 2.9%	NR
Retrospective (66)	WBRT before Pembro. (8/30)	SRT before Pembro. (13/30)	Pembro. (9/30)	Asymptomatic	87.5% vs. 46.2% vs. 66.7%	87.5% vs. 38.5% vs. 66.7%	iPFS: 7.1m vs. 4.8m vs. nr PFS: 7.1m vs. 3.5m vs. 10.2m	NR	37.5% vs. 23% vs. 11.1%

NSCLC, Non-Small Cell Lung Cancer; BMs, Brain Metastases; AEs, Adverse Events; N/A, Not Applicable; NR, Not Reported; nr, Not Reach; HR, Hazard Ratio; ICI, immune checkpoint inhibitor; RT, radiotherapy; SRT, stereotactic radiation therapy; Pembro., Pembrolizumab; Nivo., Nivolumab; OS, Overall Survival; PFS, Progression-free Survival; LCR, local control rate; iPFS, Intracranial Progression-free Survival.

While the advantages of radiotherapy combined with immunotherapy are promising, three concerns remain for clinicians. The primary concern is safety. In recent years, an increased incidence of radionecrosis was reported when applying ICIs in patients undergoing SRT for BMs. Radionecrosis significantly impacts the quality of life, leading to focal neurologic deficits, headaches, nausea, and seizures. A retrospective research has shown that receipt of immunotherapy was associated with symptomatic radionecrosis after adjustment for tumor histology (HR=2.56); this association was stronger in BMs patients from melanoma(n=145) (HR=4.02) (67). The outcome of NSCLC with BMs was not published. There were also more recent studies suggesting that ICIs were not associated with the increasing incidence of radionecrosis in NSCLC-BMs (64). This discordance may be attributed to histology heterogeneity and difficulty to distinguish radionecrosis from tumor progression in radiology. Hence, more studies are needed to clarify the association between immunotherapy and radionecrosis. Another major concern is the optimal timing of combining radiation and immunotherapy. Based on the aforementioned reciprocal mechanisms, some investigators have proposed that immunotherapy should be administered after radiotherapy so that the reciprocal effect could be maximized while activated immune cells avoid being damaged by radiation (68). Many retrospective investigations observed better outcomes in RT+ICI. However, they did not limit the order of between RT and ICI management, and the intervals between these two managements varied widely, ranging from two weeks to three months (64, 65, 69). A meta-analysis showed that concurrent SRT with ICI performed better OS than sequential therapy in the treatment of NSCLC patients with BMs (HR=0.39), but there were only two studies involved (70). In addition, the radiation dose is also attracting attention. Some investigations considered that hypofraction radiotherapy (HFRT) or SRT might better activate anti-tumor immune responses and preserve lymphocytes than traditional radiotherapy (71–73). However, there were also retrospective data showing that WBRT plus ICI performed better than SRT plus ICI in the treatment of NSCLC patients with BMs (66). Therefore, prospective trials are needed to determine the optimal dose-fraction scheme.

### ICI Combined With Chemotherapy

Like radiotherapy, there are also synergy effects between immunotherapy and chemotherapy, which is the theoretical basis of combination (74, 75). Recently, numerous prospective clinical evidence has confirmed the advantages of this combination (**Table 3**).

**Table 3:** Clinical investigations of ICI and other systematic therapies combination in the treatment of NSCLC patients with BMs.

Type of combination	Data sources	Arm (patients with BMs/all patients)		BMs Conditions	Response (ORR)		Survival	Safety (≥3 Grade AEs)	
		Experimental	Control		CNS	Systematic		CNS	Systematic
ICI + CT	Prospective (76)	Pembro. + CT (73/410)	Placebo+ CT (35/206)	Nonsquamous; Asymptomatic; Untreated;	NR	NR	PFS=6.9m vs. 4.7m, HR=0.42; OS=19.2m vs. 7.5m, HR=0.41;	NR	80% vs. 63.6%
	Prospective (77)	Pembro. + CT (105/748)	CT (66/550)	Asymptomatic;	NR	39.0% vs. 19.7%	PFS: 6.9m vs. 4.1m, HR=0.44; OS: 18.8m vs. 7.6m, HR=0.48	32.4% vs. 17.2%*	59.8% vs. 45.3%
	Prospective (78)	Camre. + CT (11/205)	CT (6/207)	Nonsquamous; Asymptomatic; CT-naive;	NR	NR	PFS: HR=0.14	NR	NR
	Prospective (79, 80)	Sinti. + CT (36/112)	Placebo + CT (22/86)	Nonsquamous; Asymptomatic; Untreated;	NR	NR	PFS: HR=0.491; OS: HR=0.565	NR	NR
	Prospective (81)	Toripa. + CT (6/40)	N/A	Asymptomatic; With EGFR mutations but T790M; Failed from prior TKI;	NR	66.7%;	NR	NR	NR
	Prospective (82)	Atezo. + CT (40)	N/A	Nonsquamous; Untreated;	40%	47.5%	iPFS: 6.9m; PFS: 8.9m; OS: 13.6m	NR	55%
ICI + AAT	Prospective (83)	ABCP (28/400)/ ACP (48/402)	BCP (24/400)	Nonsquamous; Asymptomatic; Treated;	NR	NR	iTTD: HR=0.68 for ABCP vs. BCP; HR=1.55 for ACP vs. BCP	NR	64.3% vs. 35.4% vs. 41.7%
	Prospective (84)	Sinti. + Anlotinib (4/22)	N/A	Asymptomatic; Systemic therapy-naive	100%;	75%;	1y-PFS: 50.0%; 1y-OS: 100%	NR	NR
ICI + ICI	Prospective (85)	Nivo. + Ipili. (69/583)	CT (66/583)	Asymptomatic; Treated;	NR	33% vs. 26%;	PFS: 5.4m vs. 5.8m, HR=0.79 OS: 18.8m vs. 13.7m, HR=0.57;	46% vs. 42%*	NR
	Prospective (86)	Nivo. + Ipili. + CT (65/361)	CT (58/358)	Asymptomatic; Treated;	NR	NR	OS: 19.9m vs. 7.9m, HR=0.47	NR	NR

NSCLC, Non-Small Cell Lung Cancer; BMs, Brain Metastases; AEs, Adverse Events; N/A, Not Applicable; NR, Not Reported; nr, Not Reach; HR, Hazard Ratio; CT, chemotherapy; AAT, anti-angiogenic therapy; Pembro., Pembrolizumab; Nivo., Nivolumab; Atezo., Atezolizumab; Camre., Camrelizumab; Sinti., Sintilimab; Toripa., Toripalimab; ABCP, Atezolizumab + Bevacizumab + Carboplatin+ Paclitaxel; ACP, Atezolizumab + Carboplatin+ Paclitaxel; BCP, Bevacizumab + Carboplatin+ Paclitaxel; OS, Overall Survival; PFS, Progression-free Survival; ORR, Objective Response Rate; iPFS, Intracranial Progression-free Survival; iTTD, Intracranial Time to Development (of New Brain Metastases).

*Any grade AEs.

Synergies between chemotherapy and ICIs yield to NSCLC patients with BMs in prolonged survival, including PFS and OS. This benefit has been observed in the KEYNOTE-189 study of pembrolizumab and chemotherapy vs. chemotherapy alone (76). The HR for PFS was 0.48 in the overall population, 0.48 in patients without metastases, and 0.42 in patients with BMs, and the HR for OS was 0.56, 0.59, 0.41 in the three groups above, respectively. Particularly, compared with chemotherapy alone, OS was observed a remarkable prolongation in pembrolizumab combined with chemotherapy (19.2 months vs. 7.5 months). Similar conclusions were supported in a pooled analysis of KEYNOTE-021, 189, 407 (77), including 171 NSCLC patients with asymptomatic BMs in 1298 advanced NSCLC. Systematic ORR in the combination arm was also significantly enhanced as compared with the chemotherapy alone arm (39.0% vs. 19.7%). Notably, for NSCLC patients with BMs, the magnitude of benefit from ICIs combined with chemotherapy seems to be realized regardless of PD-L1 expression. In addition, for those advanced NSCLC patients with EGFR mutation who failed from prior first-line targeting therapy, ICIs and chemotherapy combination may work well, though observed only in the trial with a small sample (81). Collectively, adding ICIs into chemotherapy can significantly improve survival for NSCLC patients with BMs. Relevant prospective clinical studies are listed in **Table 3**.

Like radiation therapy, the sequence and dose of chemotherapy combined with ICI also make a difference in the efficacy of treatment (87). Zhu et al. found docetaxel delivery before anti-PD-1 ICI with an interval of two days could initiate a more powerful anti-tumor response than simultaneous delivery and post delivery in multiple tumor models (88). However, patients in large randomized clinical trials are almost administered with chemotherapy drugs and ICI on the same day, followed by ICI maintenance (with or without chemotherapy), probably due to patient compliance. To date, no consensus has been reached regarding the dose and sequence strategies used in combinational cancer immunotherapies. How to optimize the scheme of administration relative to each kind of drug remains to be studied.

### ICI Combined With Anti-Angiogenic Agents

It is commonly believed that anti-angiogenic agents limit tumors growth by inhibiting the tumor vasculature. Nevertheless, a low dose of anti-angiogenic agent may instead induce the normalization of abnormal tumor vessels, decreasing tumor-promoting hypoxia and increasing accessibility for immune cells and other therapeutic agents to reach the TME, which facilitate the efficacy of immunotherapy (89).

In IMpower150 trial (83), patients were randomized 1:1:1 to receive atezolizumab + bevacizumab + carboplatin/paclitaxel (ABCP), atezolizumab + carboplatin/paclitaxel(ACP), or bevacizumab + carboplatin/paclitaxel (BCP). With a good tolerance, significantly improved PFS and OS were observed in the ABCP group compared with the BCP group for metastatic nonsquamous NSCLC, regardless of PD-L1 expression and EGFR or ALK genetic alteration status. Outcomes from the latest IMpower150 exploratory analyses in the subgroup with BMs indicated that the ABCP regimen could delay the time to development of new BMs (HR=0.68) for ABCP versus BCP and 1.55 for ACP versus BCP). A phase 1b trial assessed sintilimab (a PD-1 inhibitor) combined with anlotinib (a multi-target tyrosine kinase inhibitor with anti-angiogenic action) in the frontline setting for advanced NSCLC (84). This chemotherapy-free regimen presented encouraging efficacy, durability, and safety profile regardless of PD-L1 expression. Notably, all four involved patients with asymptomatic BMs at baseline achieved intracranial complete response (CR), and three of them achieved overall partial response (PR), indicating that sintilimab plus anlotinib had synergistic effects in the brain. The outcomes of a further trial are worth expecting.

The primary concern for this combination is still safety. In the BMs subgroup of Impower150 trial (83), the ABCP group had the highest incidence of Grade 3~4 trAEs among the three groups. Besides, treatment withdrawal due to AEs occurred in 42.9% of patients in the ABCP arm. On the plus side, there were no Grade 5 AEs with ABCP. Additionally, an understandable concern for AEs is intracranial hemorrhage, which should be highly regarded, although the management of anti-VEGF regents may make no contribution to an increased risk of intracerebral hemorrhage in NSCLC patients (90). Due to the lack of higher-level evidence, it is necessary to closely monitor the risk of intracerebral hemorrhage in patients with BMs when using ICIs plus anti-angiogenic agent regimens. Another imperative concern in clinical practice is the cost. After all, the promising regimen in Impower150 trial involves four drugs. A cost-effectiveness analysis from the United States showed that the incremental cost-effectiveness ratio for ABCP was $568,967 per quality-adjusted life-year (QALY) compared with BCP and $516,114 per QALY compared with CP (91). The issue of cost should be taken seriously by all parties.

### Multiple ICIs Combination

Blocking multiple immune checkpoints seems natural to activate anti-tumor immunity to a greater extent. Nivolumab plus ipilimumab have been approved for metastatic melanoma and NSCLC by FDA (92), but the failure of durvalumab plus ipilimumab gave us a warning that not all double-ICIs regimens worked well. Despite the fact that all ICIs play roles in releasing brakes that limit the immune system, the specific mechanisms for reviving anti-tumor immunity are peculiar. For instance, circulatory and resident T cells are subject to blocking the PD-1/PD-L1 axis, whereas lymphocytes in lymph nodes are subject to be activating by CTLA-4 inhibitors (93). Furthermore, different ICIs may function on their preferred subsets of T cells (94). Therefore, it is rational that the combination of dual ICIs may produce spatio-temporal synergies.

Checkmate-227 part 1 evaluated nivolumab plus ipilimumab versus chemotherapy in the treatment of advanced NSCLC. A *post-hoc* analysis (85) demonstrated that the double-ICIs arm presented higher ORR (33% vs. 26%), with a longer duration of response (24.9 months vs. 8.4 months). Longer OS was observed in the double-ICIs arm (18.8 months vs. 13.7 months). The rate of any-grade CNS AEs was 46% in BMs patients treated with double-ICIs, most of which were Grade 1~2, while it was 42% for those treated with chemotherapy. Chemotherapy or radiotherapy can be added into a double-ICIs regimen. Nivolumab + ipilimumab + two-cycle chemotherapy has been proved to have an advantage over four-cycle chemotherapy in the BMs subgroup of Checkmate-9L trial (86). Of note, double-ICIs combined with short-course chemotherapy eliminated the cross point of OS curve in Checkmate-027, which demonstrated the advantage of the combination strategy. Concurrent/sequential SBRT combined with nivolumab and ipilimumab was well tolerated (95). Multimodality therapy is valued to achieve durable metastases control and survival (**Table 3**).

## Challenges and Perspectives

**Figure 3:**
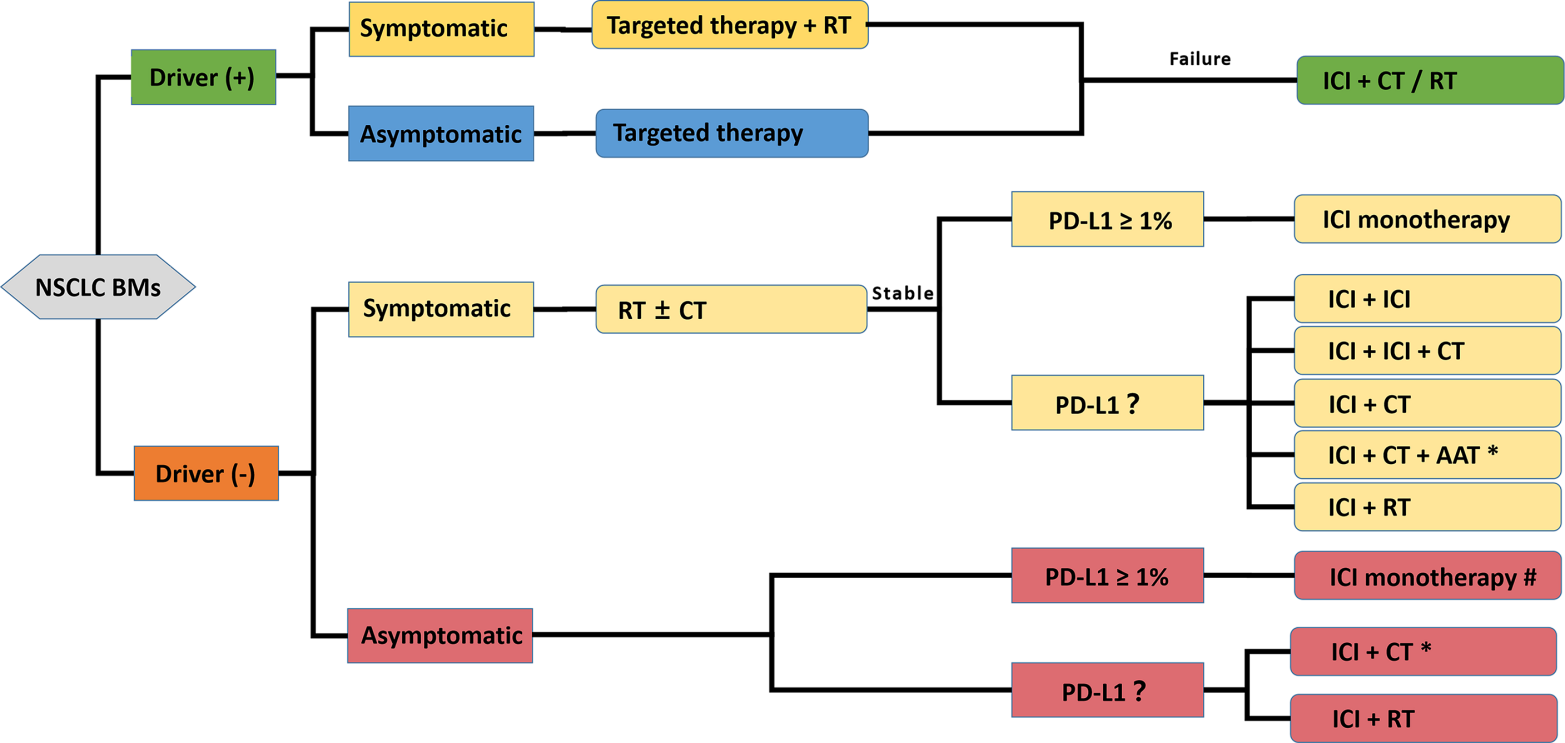
A propositional management algorithm for NSCLC patients with BMs who are candidates for ICI-containing comprehensive treatment. This algorithm considers patients who are candidates for an ICI-containing comprehensive therapy after the standard patient examination and tumor specimen evaluation (driver-gene status and PD-L1 expression evaluation). Targeted reagent-centered therapy should be recommended first for driver-positive patients owing to a favorable intracranial response rate. Considering the poor prognosis of BMs, ICI monotherapy is recommended with more caution, and ICI-containing combination therapy is encouraged. ICI Patients with symptomatic or multiple BMs could be treated with RT strategies. The final therapeutic decision should be made by a multidisciplinary tumor board. * only appropriate for nonsquamous NSCLC; # optimal when PD-L1 ≥ 50%; ICI, Immune Checkpoint Inhibitor; CT, Chemotherapy; RT, Radiotherapy; TT, Targeted therapy; AAT, Anti-angiogenic therapy.

Based on the basic researches and clinical data presented in this review, sufficient evidence exists to support the continued exploration of the novel value of immunotherapy for NSCLC BMs. An ICI-containing algorithm in the management of NSCLC-BMs is presented in **Figure 3**. However, it must be noted that until more robust clinical trials are conducted, NSCLC-BMs patients should be individually evaluated by multidisciplinary tumor boards in highly experienced centers. Also, several considerations need to be adequately addressed before the development of a clinical trial designed to widely test the setting.

### Patient Selection

In the era of immunotherapy, the appropriate patient selection remains of paramount importance. In NSCLC BMs, prognosis significantly depends on several factors, including age, Karnofsky performance status, extracranial metastases, number of BMs, and the presence of driver-gene mutations. Together, these clinical parameters constitute lung-graded prognostic assessment (lung-GPA), the most established tool to estimate survival in lung cancer. GPA of 4.0 and 0.0 correlate with the best and worst prognosis, respectively, with OS varying widely from 7 months to 47 months (96). Some studies have shown that some clinical parameters, including but not limited to those involved in GPA, are associated with intracranial outcomes and survival in ICIs treatment of NSCLC BMs (11, 97). As noted previously, the benefit acquired from ICIs in NSCLC patients with asymptomatic BMs is likely to have no different from that in patients without BMs. Hence, we recommend that NSCLC patients with asymptomatic BMs are not supposed to be excluded routinely from clinical trials on ICIs. Symptomatic BMs often have poor efficacy and outcome. Besides active BMs per se, the decline in the efficacy of immunotherapy is attributed to steroids, which are considered immune suppressors and routinely used to control intracranial symptoms and modify side effects of other therapies in patients with BMs (14, 98). Moreover, harboring driver-gene mutation, indicating a favorable prognosis, is associated with low benefits from ICIs (99). Hence, GPA in the setting of immunotherapy may need to be updated.

Developing reliable biomarkers is an important approach to accurately select patients. Currently, for anti-PD-1/PD-L1 treatment, PD-L1 remains the most commonly used stratified biomarker in both clinical practice and trials. A study demonstrated that PD-L1 expression might predict OS in NSCLC BMs patients receiving immunotherapy. Importantly, it was independent of lung-GPA (100). It also noted that intracranial PFS did not show an association with PD-L1 expression (100). However, the specimens for testing PD-L1 expression in almost all studies are from primary lesions. As mentioned earlier, PD-L1 expression is at variance between BMs and primary lesions (30, 31). Therefore, whether PD-L1 can be a robust marker for ICIs intracranial response remains to require further investigations. Besides PD-L1, tumor mutational burden (TMB) is also approved as a biomarker of ICIs therapy, such as pembrolizumab-based therapy and nivolumab + ipilimumab combination therapy, in pan-cancer (101–103). Remarkably, TMB is site-specific in NSCLC and is highest in lung adenocarcinoma BMs (104). Beyond PD-L1 and TMB, emerging genomic biomarkers for immunotherapy are under development (105, 106).

A major restriction of studying the tumor immune microenvironment of BMs is that it is extremely challenging to obtain intracranial specimens. Therefore, simple substitutions are required. Liquid biopsy technique based on cerebrospinal fluid provides the opportunity to precisely acquire and monitor BMs in real-time and guide immunotherapy. Cell-free DNA (ct-DNA) and immune cell RNA profiling of CFS enable to characterize genomic information and immune cells infiltration of BMs and predict prognosis eventually (107–110). The presence of circulating tumor cells (CTCs) has been reported to be associated not only with NSCLC recurrence and metastasis but also with worse tumor response to ICI (111). Methods have been developed to characterize CTCs of NSCLC-BMs in CSF (112). Further work is needed to confirm the potential value of CTCs in predicting the efficacy of ICI for NSCLC-BMs. Besides liquid biopsy, advanced imaging techniques and artificial intelligence in radiomics will bring about a revolutionary shift in predicting cancer outcomes (113). For instance, the deep learning models from CT (114) or PET-CT (115) provide a noninvasive method to predict high PD-L1 expression of NSCLC and infer clinical outcomes in response to immunotherapy.

With the digitalization of radiology, histopathology, genomics and clinical information, it is necessary to integrate and analyze these data, because none of them can fully characterize tumors alone. In other words, they are complementary. For example, radiological scans and pathology specimens describe tumors spatially at different dimensions. However, at present, even when these data are available, they are rarely integrated. Artificial intelligence and deep learning provide an opportunity for multimodal data integration (116). One example is that, by integrating PET-CT imaging, RNA-sequencing, and histology, differential immuno-metabolic crosstalk in lung squamous cell carcinoma and adenocarcinoma was observed (117). Another study found that the combination of features from histological imaging and MRI outperformed unimodal classifiers for the stratification of brain tumor subtypes (118). BMs possess the intricate characteristics of both primary solid tumors and neurological tumors. Thus, the integration of information may be a promising direction for developing reliable biomarkers for BMs.

### Response Assessment

The response assessment of BMs depends not only on changes in the size of the targeted lesions but also on changes in neurological status and steroid dosage. Previous measures, such as tumor shrinkage rate and survival time, to evaluate the efficacy of immunotherapy are not comprehensive and are not able to imply early efficacy because some lesions have a temporary pseudoprogression and then respond well. Published studies have reported a wide range of incidences of pseudoprogression (from less than 1% to more than 20%) (119–121). Regardless, consequences with failure to identify pseudoprogression are substantive and undesired, including premature discontinuation of an effective therapy and overestimating the efficacy of a subsequent therapy. Therefore, with the shift of the management of BMs in the era of immunotherapy, understanding of the response assessment of BMs needs renovation.

**Figure 4:**
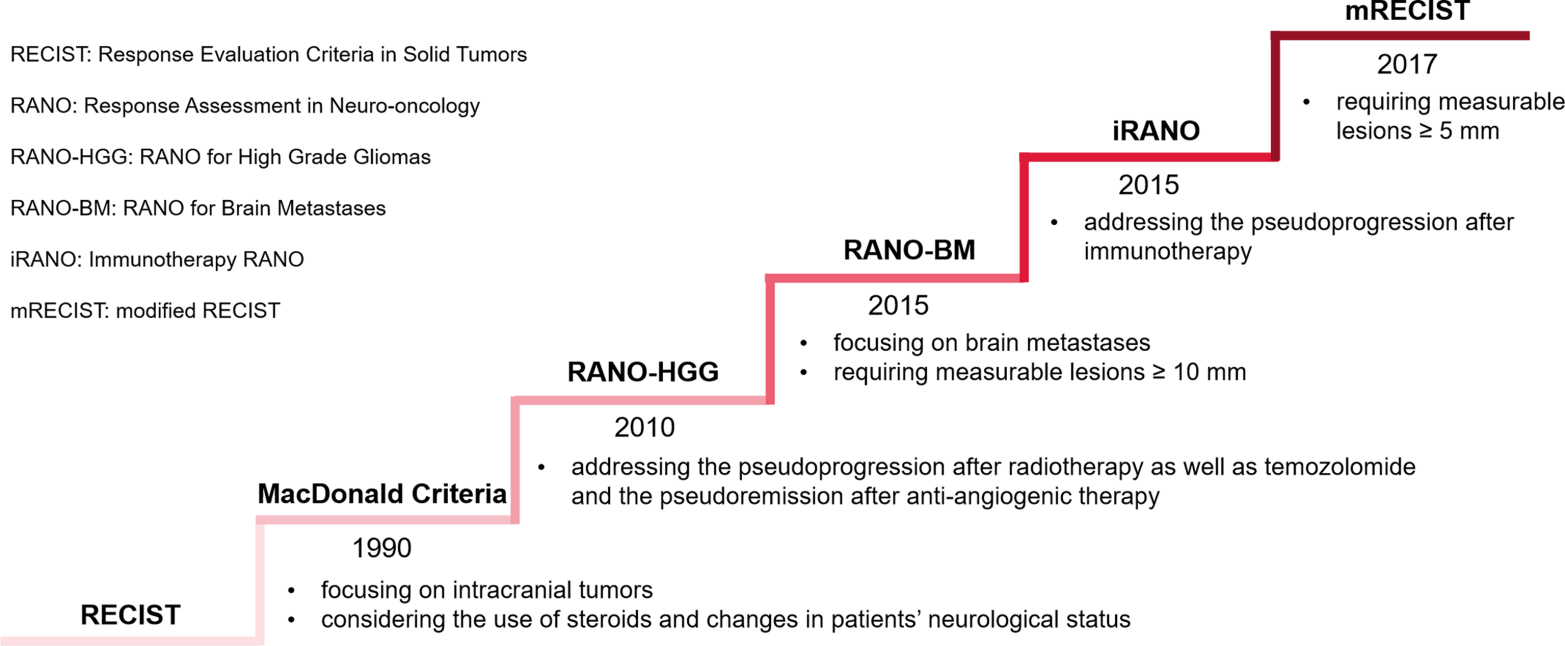
Evolving criteria of response assessment to BMs. The ladder rises by the time a standard is first released rather than its popularity.

Firstly, the criteria of response assessment to BMs are constantly evolving (**Figure 4**). Most solid tumors were evaluated by Response Evaluation Criteria in Solid Tumors (RECIST) 1.1. Given the use of steroids and changes in patients’ neurological status, MacDonald Criteria was established, primarily applied to gliomas (122). Subsequently, to cope with the challenges posed by the pseudoprogression after radiotherapy as well as the pseudoremission after anti-angiogenic therapy, the Response Assessment in Neuro-Oncology (RANO) working group published the Response Assessment in Neuro-Oncology for High-grade Gliomas (RANO-HGG) (123). Although MacDonald Criteria and RANO-HGG could be extended to BMs, BMs possess the characteristics of both primary solid tumors and neurological tumors. Hence, for high-quality assessment of BMs, the RANO working group established RANO for BMs (RANO-BM) (124). The RANO-BM uses a one-dimensional method to measure tumor size, requiring measurable lesions to be at least 10 mm in diameter, allowing up to 5 lesions to be targeted, and incorporates the patient’s performance status and steroid use as a basis for evaluation when determining disease remission or progression. Qian et al. proposed modified RECIST (mRECIST) 1.1 criteria to adapt to the application of immunotherapy in the treatment of BMs (125). The standard eased the restriction of measurable lesion length to ≥5 mm, allowing more patients to be included in clinical studies. Immune-related Response Criteria (irRC) pointed out that if there was no significant decrease in the patient’s clinical performance status, progression could not be determined by an increase in the volume of an early lesion or the appearance of a new lesion unless subsequent imaging tests could confirm (126). Based on irRC, the RANO working group developed Immunotherapy RANO (iRANO) for patients with neurologic tumors who received immunotherapy, including BMs (127). It recommended that immunotherapy for six months was required in patients without clinical response. Patients with radiological progression should undertake a radiological follow-up after three months and clinicians should compare the two images to review post-treatment outcomes. iRANO is proposed to address the potential pseudoprogression after immunotherapy, with a 6-month window to assess true response, avoiding premature discontinuation of treatment in patients who are likely to benefit from immunotherapy potentially. However, iRANO has not yet been widely manipulated in clinical trials and practice because of its complicated implementation, which limits the popularity of iRANO to a certain extent.

Similarly, advanced tools are emerging to facilitate response assessment. An example is that magnetic resonance (MR) spectroscopy and perfusion might increase the accuracy of differentiating recurrent tumors from radionecrosis in patients with gliomas or BMs (128). Of particular interest are radiolabeled amino acids for brain tumor imaging using positron emission tomography (PET) because of their increased uptake in neoplastic tissue but low uptake in the normal brain parenchyma (129), which allows the accurate depiction of BMs to delineate BMs extent, assess treatment response, and differentiate treatment-related changes from tumor progression (130). Recently, this imaging technique was strongly recommended by the RANO working group (131, 132). Other tracers, such as radiolabeled analog to the nucleoside thymidine, were developed to assess cellular proliferation and may be of great value in the differentiation of BMs pseudoprogression after immunotherapy (133).

Importantly, on the basis of more investigations, more consensus needs to be reached in the context of the existence of various criteria and assessment tools.

## Conclusion

Advances in ICIs have resulted in the management of BMs patients from NSCLC navigating toward the immunotherapy era. ICI monotherapy and combination have embodied novel value in enhancing intracranial response, prolonging survival, delaying BMs, and improving quality of life. In summary, the activity of ICIs for the treatment of NSCLC BMs should not be drastically underestimated, especially for selected patients. Considering the poor prognosis of BMs as well as the reciprocity between immunotherapy and other therapies, the synergistic combination treatment is promising. Critically, before the extensive application of this combination protocol in clinical practice, more preclinical and clinical trials are urgently needed to provide definite evidence and resolve the challenges discussed above.

## Author Contributions

GY contributed to the original draft, investigation, and resources. LX contributed to review, and funding acquisition. XS contributed to study design, review, and funding acquisition. All authors read and approved the submitted manuscript.

## Funding

The study was funded by the National Natural Science Foundation of China (No. 82172866).

## Conflict of Interest

The authors declare that the research was conducted in the absence of any commercial or financial relationships that could be construed as a potential conflict of interest.

## Publisher’s Note

All claims expressed in this article are solely those of the authors and do not necessarily represent those of their affiliated organizations, or those of the publisher, the editors and the reviewers. Any product that may be evaluated in this article, or claim that may be made by its manufacturer, is not guaranteed or endorsed by the publisher.

## Abbreviations

NSCLC, Non-small cell lung cancer; BMs, Brain metastases; EGFR, Epidermal growth factor receptor; ALK, Anaplastic lymphoma kinase; PD-1, Programmed cell death-1; PD-L1, Programmed cell death ligand-1; CTLA-4, Cytotoxic T-lymphocyte antigen-4; ICI, Immune checkpoint inhibitor; mAb, Monoclonal antibody; RT, Radiotherapy; SRT, Stereotactic radiotherapy; WBRT, Whole-brain radiotherapy; HFRT, Hypofraction radiotherapy; CT, Chemotherapy; Computed tomography; AAT, Anti-angiogenic therapy; BBB, Blood-brain barrier; CSF, Cerebrospinal fluid; TME, Tumor microenvironment; TILs, Tumor-infiltrating lymphocytes; TAMs, Tumor-associated macrophages; Tregs, Regulatory T cells; MDSCs, Myeloid-derived suppressor cells; VEGF, Vascular endothelial growth factor; VEGFR, Vascular endothelial growth factor receptor; VCAM-1, Vascular cell adhesion molecule-1; ICAM-1, Intercellular adhesion molecule 1; INF, Interferon; CNS, Central never system; HR, Hazard ratio; CI, Confidence interval; CR, Complete response; PR, Partial response; ORR, Objective response rate; OS, Overall survival; PFS, Progression free survival; iPFS, Intracranial progression-free survival; iTTD, Intracranial time to development; AEs, Adverse events; trAEs, Treatment-related adverse events; QALY, Quality-adjusted life-year; FDA, Food and Drug Administration; lung-GPA, Lung-graded prognostic assessment; Ct-DNA, Circulating tumor DNA; CTCs,irculating tumor cells; RECIST, Response Evaluation Criteria in Solid Tumors; RANO, Response Assessment in Neuro-oncology; RANO-HGG, RANO for High Grade Gliomas; RANO-BM, RANO for Brain Metastases; iRANO, immunotherapy RANO; mRECIST, modified RECIST; PET, Positron emission tomography; MR, Magnetic resonance.

## Glossary

**Table:**

NSCLC	Non-small cell lung cancer
BMs	Brain metastases
EGFR	Epidermal growth factor receptor
ALK	Anaplastic lymphoma kinase
PD-1	Programmed cell death-1
PD-L1	Programmed cell death ligand-1
CTLA-4	Cytotoxic T-lymphocyte antigen-4
ICI	Immune checkpoint inhibitor
mAb	Monoclonal antibody
RT	Radiotherapy
SRT	Stereotactic radiotherapy
WBRT	Whole-brain radiotherapy
HFRT	Hypofraction radiotherapy
CT	Chemotherapy, Computed tomography
AAT	Anti-angiogenic therapy
BBB	Blood-brain barrier
CSF	Cerebrospinal fluid
TME	Tumor microenvironment
TILs	Tumor-infiltrating lymphocytes
TAMs	Tumor-associated macrophages
Tregs	Regulatory T cells
MDSCs	Myeloid-derived suppressor cells
VEGF	Vascular endothelial growth factor
VEGFR	Vascular endothelial growth factor receptor
VCAM-1	Vascular cell adhesion molecule-1
ICAM-1	Intercellular adhesion molecule 1
INF	Interferon
CNS	Central never system
HR	Hazard ratio
CI	Confidence interval
CR	Complete response
PR	Partial response
ORR	Objective response rate
OS	Overall survival
PFS	Progression free survival
iPFS	Intracranial progression-free survival
iTTD	Intracranial time to development
AEs	Adverse events
trAEs	Treatment-related adverse events
QALY	Quality-adjusted life-year
FDA	Food and Drug Administration
lung-GPA	Lung-graded prognostic assessment
Ct-DNA	Circulating tumor DNA
CTCs	Circulating tumor cells
RECIST	Response Evaluation Criteria in Solid Tumors
RANO	Response Assessment in Neuro-oncology
RANO-HGG	RANO for High Grade Gliomas
RANO-BM	RANO for Brain Metastases
iRANO	immunotherapy RANO
mRECIST	modified RECIST
PET	Positron emission tomography
MR	Magnetic resonance
